# Diseases of the Kidney

**Published:** 1905-03-04

**Authors:** 


### DISEASES OF THE KIDNEY.

Excretion of Sodium Chloride.—It appears that in some cases of Bright's disease there is a loss of the power to excrete sodium chloride, which accumulates in the tissues and may cause œdema. Horder [1] points out that a healthy man takes about half an ounce of common salt daily, while the kidneys can excrete an ounce or more; but in disease they may not excrete more than 30 grains. If we exclude all use of salt as a condiment and in cooking there will still be 15 to 22 grains from the food in ordinary mixed diet. When œdema is present an increase in the œdema and the weight of the patient will often occur on the addition of more or less salt to this minimum, and if we then reduce the salt the œdema will disappear. On the other hand, in certain cases of interstitial nephritis more salt may be excreted than is taken, and here the prognosis is grave. Manté and Claude, too, show that when the excretion of salt is seriously damaged a pure milk diet is necessary. In some cases of hepatic cirrhosis, too, there is a failure to excrete salt, and the same thing has been found in pleurisy, white leg, and tubercular peritonitis. Some degree of improvement in these affections has been obtained by reducing the salt taken. From these observations we learn why in some cases a milk diet has such beneficial results.

**Kidney Extract.**—Renaut and Dubois[2] report remarkable improvement by treatment with an extract of fresh pig's kidney. A couple of fresh kidneys are chopped fine and washed with water to remove any urine present in the tubes. They are then pounded with half a pint of water and a little salt, and left for four hours. The clear liquid is decanted off, and given to the patient during the day in three or four doses. A little soup may be added to make it more palatable, but it must not be overheated. The treatment should be continued for some time, with breaks of a few days in each fortnight. No ill-effects have been noted.

**Excretion of Methylene Blue.**—The elimination of *methylene blue* has been regarded by some as giving little or no evidence of the actual disease present. Indeed, as in the case of sodium chloride, there may be in certain persons a failure to eliminate the blue, which is an inadequate measure of the nephritis. Generally in chronic Bright's disease the excretion is delayed. Achard and Paisseau[3] seem, however, to show that methylene blue is a measure of the excretion of urea both in health and disease. Widal thinks that there is no delay of excretion in tubal nephritis.

**Treatment of Nephritis.**—Beverley Robinson[4] in acute nephritis advises some form of milk diet, if possible, at first, and then amylaceous food and water in abundance, hot wet packs, high rectal injections of hot water after the bowels have been freely opened; in uræmia nitroglycerine, bleeding and saline injections, and oxygen. The diarrhœa of chronic Bright's disease will sometimes yield to prolonged hot saline injections. As to morphia in uræmic states, while this writer is generally opposed to it he thinks he has seen benefit when the pupils are dilated or at least normal in size, and suggests that bleeding and saline transfusion might well be employed at once after morphia has been given to avoid danger. Theobromine appears to be a valuable diuretic, and to possess some power of diminishing the albumen in the urine. Petitti[5] in speaking of agurin, a new diuretic, remarks that when œdema is present, the safest treatment is prolonged drainage, whilst the use of sudorifics, diuretics or purges may lead to uræmic attacks. He had two instances of this when giving agurin, which, however, caused powerful diuresis. It is otherwise well borne by the patients; the usual dose is 23 to 45 grains a day and the increased diuresis commences from the first day of the administration and the amount of urea chlorides and phosphates excreted rises rapidly. In cardiac affections it often acts better than digitalis.

[1] Pract., Nov. [2] Ibid. [3] Birm. Med. Rec., Sept. [4] Amer. Jour., Med. Sc., p. 55, vol. ii. [5] Clin Excerpts, Jan. 1905.

*(To be continued.)*

